# Ageing Renal Patients: We Need More Collaboration between Geriatric Services and Nephrology Departments

**DOI:** 10.3390/healthcare3041075

**Published:** 2015-10-30

**Authors:** Helen Alston, Aine Burns

**Affiliations:** Centre for Nephrology, Royal Free Hospitals NHS Foundation Trust, Pond Street, London NW3 2QG, UK; E-Mail: aine.burns@nhs.net

**Keywords:** geriatrics, nephrology, multidisciplinary working, patient-centred care, quality of life

## Abstract

There has been a significant increase in the number of frail older patients diagnosed with advanced chronic kidney disease (CKD) over the past thirty years. These elderly patients have high levels of comorbidity, and as a consequence the face of renal medicine is changing—There is an increasing need to focus on traditionally geriatric areas of expertise such as falls prevention and rehabilitation, and to shift our emphasis onto improving patient well-being rather than longevity. Over the past decade, many nephrologists have found that they are already acting as de facto “amateur geriatricians”. This denies patients both the benefits of specialist geriatric assessment, and equally importantly denies them access to the wider geriatric multidisciplinary team. This article describes the prevalence and underlying causes of the so-called “Geriatric Giants” in patients with advanced CKD, and discusses possible improvements in care that closer working with geriatricians could bring.

## 1. Introduction

There has been a significant expansion in the number of frail older patients diagnosed with advanced chronic kidney disease (CKD) over the past thirty years [1]. Indeed, the burden of CKD disproportionately affects older patients. Despite this, most older patients die with CKD (due to other comorbidities), not from it [2,3,4,5,6]. These elderly patients have high levels of comorbidity, and as a consequence the face of renal medicine is changing—There is an increasing need to focus on traditionally geriatric areas of expertise such as falls prevention and rehabilitation, and shift our emphasis onto improving patient well-being rather than longevity.

In the past, initiatives aimed at improving links between renal medicine and geriatric medicine have been unable to generate sustained enthusiasm [7]. Although nephrologists have long understood the benefits of multidisciplinary working (and are used to working alongside renal dietitians and psychologists) they have been slower to appreciate the expertise that geriatricians, physiotherapists and occupational therapists can provide. This is partly due to cultural barriers and “silo-isation” that exists between hospital departments. As a smaller specialty, most nephrology departments are based in tertiary hospitals and in these large organisations consultants from different specialties may barely know each other, much less understand the skills and services that each has to offer. This lack of knowledge works both ways—Geriatricians are often surprised to discover the breadth of case-mix managed by nephrologists, and the high levels of comorbidity and functional impairment found in our inpatients.

However over the past decade, many nephrologists have realised that they are already acting as de facto “amateur geriatricians” [7], and there has been a push to improve renal training in this area. The American Accreditation Council for Graduate Medical Education (ACGME) now includes mandatory geriatric nephrology training as part of nephrology fellowship programmes, the American Society of Nephrology and UK Renal Association and British Renal Society regularly include geriatric nephrology streams in their annual conferences, and The Kidney Disease in Older People Network (KidoPEN) was established in 2012 to share expertise and good practice in the management of older people with CKD.

Both specialties are very familiar with the concept of patient-centred care. However while renal services often focus on holistic symptom control, dietary changes and the psychological impact of renal disease, geriatric comprehensive assessment focuses on functional status, cognitive impairment and polypharmacy, and aims to maintain or improve quality of life and prevent future hospital readmissions [8,9,10,11,12]. This may also involve guiding patients towards treatment options which will maintain their independence, and initiating Advanced Care Plans to keep patients in their own homes or prevent unwanted escalations in care.

The so-called “Geriatric Giants” (frailty, falls, dementia/delirium and to a lesser extent incontinence) [13] will be extremely familiar to nephrologists in practice, although perhaps the research around their presentation in renal patients is less familiar. This article describes the prevalence and underlying causes of these Geriatric Giants in renal patients, and discusses possible improvements in care that closer working with geriatricians could bring.

## 2. Frailty and Multiple Comorbidities

Frailty, defined by Clegg *et al*. as “a cumulative decline in multiple physiological systems and impairment of homeostasis leading to increased vulnerability to external stressors” [14], is an area of great interest in current geriatric research. It is a concept which has long been widely understood as a marker of decreased physiological reserve and increased mortality by clinicians.

There are two main models of frailty—The Rockwood Frailty Index, which views frailty as an accumulation of deficits, with patients moving along a continuum from non-frail to frail [15], and the Fried Frailty Phenotype, which sees frailty as a syndrome which is either present or absent [16], based on the presence of three or more of the following criteria: unintentional weight loss, self-reported exhaustion, weakness, slow walking speed, and low physical activity. In both models, this cumulative decline depletes homoeostatic reserves until minor stressor events trigger disproportionate changes in health status (for example, a minor urinary tract infection may lead to delirium and bedfastness in a frail patient, while the same illness would have minimal effect on a robust patient of the same age).

A high proportion of renal patients are frail. Indeed, some studies have shown that over 78% of dialysis patients aged over 70 would meet the Fried frailty criteria [17,18,19,20], compared with 6.9% of Fried’s original cohort of older patients with normal renal function [21]. There are a number of possible reasons for the extremely high proportion of frail dialysis patients—in a later study, Fried found an association between protein-energy wasting and both impaired renal function and chronic inflammation [22]. This may be exacerbated by poor intake due to uraemia and dietary restrictions. High levels of comorbidities, frequent infections and regular hospital admissions in this population will also predispose to frailty. This has a number of implications for nephrologists—should we screen for frailty, if it is so prevalent? And how should we intervene when we find it [8]?

The intervention for frailty with the strongest evidence base is the comprehensive geriatric assessment (CGA), defined as “a multidimensional interdisciplinary diagnostic process focused on determining a frail older person’s medical, psychological and functional capability in order to develop a coordinated and integrated plan for treatment and long term follow up” [23]. The resources are not currently in place to allow a comprehensive geriatric assessment for 80% of our prevalent dialysis patients, but pilot studies by Jassal and others which offered CGA to older new starters on dialysis have shown promising early results [24,25,26]. Another option might be the wider use of “Cause for Concern” screening tools to identify the failing dialysis patient and to intervene early. The British Geriatric Society’s excellent “Fit for Frailty” guidelines [27] are both comprehensive and easy to read, and are highly recommended for all clinicians who care for older patients.

## 3. Functional Status

Cook and Jassal [28] found that in a cohort of 168 prevalent haemodialysis patients aged over 65 years, only 5% were independent in all Activities of Daily Life (ADLs) and Instrumented Activities of Daily Life (IADLs), and 53% were dependent in at least one of the six basic ADLs. Almost half were unable to wash without assistance. Only a quarter could rise from a chair without using their arms, and less than a third had normal balance. Over 30% had fallen at least once in the previous year. Functional status is somewhat better in peritoneal dialysis (PD) patients [29], but this may be due to patient selection (if assisted PD is not available, only patients with relatively good functional status will be offered PD).

Kurella Tamura *et al.* [30] found that physical function declined rapidly in the three months before initiation of haemodialysis, which might be expected due to increasing uraemia. However, contrary to expectation physical function did not appear to recover following initiation of dialysis, and patients remained significantly functionally impaired. Jassal *et al.* [31] found that 30% of new starters on dialysis became more functionally dependent within the first six months of being on haemodialysis, including those patients who had previously lived independently. Although functional status did not worsen after the first six months, it did not improve either.

In contrast, Murtagh [32] found that patients managed conservatively maintained functional status until the last month of life. There is admittedly the possibility of lead-time bias here—patients were included in the study when estimated glomerular filtration rate (eGFR) was < 15mls/min (*i.e.*, long before dialysis would usually be initiated) and eGFR at point of death was not recorded, so it is quite possible that “last month of life” is equivalent to (or even earlier than) “time at which patient would have started dialysis if they had not been managed conservatively”. However Hussain *et al.* [33], a much larger study following patients from eGFR < 20mls/min, reported similar findings.

## 4. Falls and Fractures

Older dialysis patients frequently fall [34,35,36], when they do fall they are at increased risk of fractures [37,38,39,40,41], and they are more likely to have poor outcomes than similar-aged patients with normal renal function [41,42,43,44]. Cook and Jassal found that over 30% of dialysis patients aged over 65 had fallen at least once in the previous year, and only 20% of patients had normal timed functional mobility scores [28]. Post-dialysis hypotension, underlying cerebrovascular and cardiovascular disease, polypharmacy, autonomic dysfunction and peripheral neuropathy (due to co-existing diabetes and deposition of B2 microglobulin) are just some of the potential causes of these falls. There is an association between vitamin D deficiency, somatic muscular weakness and falls risk in both renal patients and the general population [35,45,46,47]. Many patients have amputations [48,49,50,51]. Many patients also have visual [52] or cognitive problems [53], and trying to climb off and onto the bed for dialysis and then to negotiate a busy, noisy dialysis unit can be a disorientating experience for many older patients. In addition, patients with cognitive problems have higher rates of falls when their attention is distracted [54,55], and dialysis units are generally rather distracting places.

Awareness of some of these problems can lead to risk reduction [41]—For example, regular medication reviews, omitting antihypertensives on the morning of dialysis to prevent hypotension, removing trip hazards in the dialysis unit and offering assistance with transfers, and early screening and referral to physiotherapy or occupational therapy for walking aids and grab rails. Simple screening tools such as the sit-to-stand test and the timed up-and-go are effective at predicting falls risk [56], and can easily be carried out in clinic or on the dialysis unit.

## 5. Cognitive Function

An estimated 30%–70% of haemodialysis patients have been shown to have some degree of cognitive impairment [53,57,58,59,60,61], and incidence of new cognitive impairment has been associated with both severity of CKD [58,62,63] and with both rate of decline of renal function and albuminuria [64]. This is probably due to a combination of comorbidity (presence of diabetes, vascular disease and hypertension are all independent risk factors [65,66]) and factors unique to CKD, such as the presence of uraemia.

Cognitive function is worse during dialysis sessions [67], presumably due to reduced cerebral perfusion, and best either just before or the day after a dialysis session. It does not seem to improve with more frequent haemodialysis [68], and there is conflicting evidence regarding improvement following transplantation [69,70,71], suggesting that some of this damage may be permanent.

Visuospatial and executive functions are disproportionately affected in these patients [65,72,73], and the NHANES III study found slower learning speeds and impaired visual concentration even in otherwise healthy, younger CKD patients (aged 20–59), compared with standardised populations [74]. This has obvious implications for pre-dialysis counselling and patient education programmes, as well as with treatment compliance. Importantly, these deficits may not be immediately obvious to clinicians in the way that loss of short-term memory or orientation might be, and will also not be detected using short screening tools such as the Abbreviated Mental Test (AMT), which is widely used to screen for dementia and delirium in patients acutely admitted to NHS hospitals.

The presence of cognitive impairment has been linked with an increased risk of hospitalisation [75] and death [60] in dialysis populations, and there are several plausible reasons for this. Firstly, the presence of comorbidities such as diabetes and vascular disease will predispose patients to both cognitive impairment and higher rates of hospitalisation and death. However, difficulties in taking medications or following dietary or fluid restrictions due to impaired executive function may also play a role.

## 6. Conclusions

As the population ages, renal patients are increasingly presenting with one of the “Geriatric Giants” alongside their nephrological diagnoses. However, their underlying renal disease mandates ongoing specialist renal input—nephrologists cannot simply abdicate responsibility for the care of these complex patients to geriatric services. Indeed few would wish to—There is a long tradition of inpatient care for dialysis and renal transplant patients being provided by nephrologists regardless of the admission diagnosis, and indeed dialysis patients often see the renal team as their first port of call for any medical problems.

These patients should already be known to renal services, so it makes obvious sense for the nephrology department to maintain responsibility for identifying patients who they feel would benefit from geriatric input, rather than simply referring everybody for assessment (of course when people are NOT known to renal services, *i.e.*, the older patient with CKD as an incidental finding, it is important to emphasise that there is good evidence that early nephrology input reduces progression [76]).

There will be some aspects of care that can only be managed by the nephrologists—For example a dialysis patient with falls due to postural hypotension may simply need their target dry weight adjusting. However the current solution, nephrologists becoming “amateur geriatricians”, denies patients both the benefits of specialist geriatric assessment, and equally importantly denies them access to the wider geriatric multidisciplinary team, including hospital at home/admission avoidance, community geriatrics, day hospitals, falls clinics, memory clinics and other specialist services. Protocols for referral to renogeriatric clinics should be developed locally to make the best use of the resources available.

From the geriatricians’ point of view, closer links with nephrology would provide expertise in the management and prevention of both CKD and acute kidney injuries (AKIs), which disproportionately affect older adults [77] and are often poorly managed [78].

Nephrology has an excellent track record of working with palliative care services to develop conservative management programmes in the UK, and with diabetologists and cardiologists to develop joint diabetic nephropathy and cardiorenal clinics respectively. The future aim for the specialty should be the establishment of joint geriatric nephrology services to ensure that our frail older patients have access to the full spectrum of services, and are not disadvantaged by their co-existing renal disease.
